# Integrating line × tester analysis and GGE biplot to identify superior sweet corn hybrids in tropical environments

**DOI:** 10.7717/peerj.20966

**Published:** 2026-05-14

**Authors:** Dedi Ruswandi, Ade Ismail, Galang Sukma Persada, Mochamad Rafi Fauzan, Fakhri Nasharul Syihab, Yuyun Yuwariah

**Affiliations:** Department of Crop Science, College of Agriculture, Padjadjaran University, Sumedang, Jawa Barat, Indonesia

**Keywords:** Combining ability, GGE biplot, G × E interaction, Sweet corn, Yield

## Abstract

Sweet corn (*Zea mays saccharata* L.) is a high-value crop for both food and industry, yet its productivity is constrained by strong genotype × environment (G × E) interactions that reduce yield stability. Developing superior hybrids requires accurate estimation of general combining ability (GCA) and specific combining ability (SCA). This study evaluated 20 inbred lines and four testers (80 hybrids) across two contrasting seasons in Garut, Indonesia, using a line × tester design and GGE biplot analysis. Significant variation was observed for all yield-related traits, with dominance effects exceeding additive effects, illustrating the value of non-additive gene action in hybrid development. GGE biplot identified L18 as the best general combiner for ear diameter and length, L2 for kernel row number, L9 for ear weight, L4 for biomass, L12 for brix, and L20 for yield. Optimal testers were T1 for brix, T2 for ear length and kernel row number, and T3 for ear weight and diameter. Superior hybrids with strong SCA included L2 × T4, L5 × T4, and L12 × T1, which consistently outperformed across seasons. This is the first comprehensive application of GGE biplot for dissecting GCA and SCA in tropical sweet corn under dual-season conditions in Indonesia. The identified parental lines and hybrids provide a valuable resource for breeding stable, high-yielding sweet corn adapted to tropical environments.

## Introduction

Sweet corn (*Zea mays saccharata* L.) represents a key vegetable crop in Asia, especially in Indonesia, where it is extensively grown both for direct consumption and for industrial uses. The crop’s market value arises from increasing consumer demand and its diverse nutritional profile, which includes macronutrients, essential amino acids, vitamins, minerals, and bioactive compounds beneficial to health (Swapna, Jadesha & Mahadevu, 2020; Baveja et al., 2022). These attributes make sweet corn a profitable commodity with expanding domestic and export markets. Consequently, enhancing the productivity and stability of sweet corn hybrids is a national priority for improving farmer incomes and meeting market demand.

Despite its importance, sweet corn yields in Indonesia remain inconsistent due to environmental fluctuations. Variations in rainfall, soil conditions, and temperature across seasons lead to pronounced genotype × environment interactions (GEI), often undermining yield consistency and adaptability. Genotypes that perform well in one season often fail in another, making the development of stable, high-yielding hybrids an urgent breeding goal.

Hybrid breeding remains an established strategy for yield enhancement, with its effectiveness relying heavily on selecting inbred lines that exhibit favorable combining ability. Line × tester analysis provides estimates of general combining ability (GCA)—reflecting additive gene action—and specific combining ability (SCA)—reflecting dominance and epistatic effects (Kempthorne, 1957; Singh & Chaudary, 1979). While GCA highlights consistently favorable parents, SCA identifies cross-specific heterotic responses.

However, environmental heterogeneity often complicates interpretation. To overcome this challenge, the genotype plus genotype × environment (GGE) biplot methodology offers a powerful graphical tool to visualize GCA, SCA, and GEI simultaneously (Yan & Tinker, 2006). Though widely applied in maize, wheat, sorghum, and lentil (Fan et al., 2007; Fellahi et al., 2013; Kannababu et al., 2017; Karimizadeh et al., 2013), its use in sweet corn remains limited, particularly under tropical conditions where seasonal variation is pronounced.

The 20 inbred lines and four testers used in this study represent diverse genetic backgrounds from the tropical sweet corn breeding program at Universitas Padjadjaran. Although formal heterotic grouping is still under development, previous trial data suggest that these lines differ substantially in sweetness profile (brix), kernel row number, ear size, and agronomic vigor. Such diversity provides an opportunity to quantify both additive and non-additive genetic effects, while the contrasting seasonal conditions of Garut allow preliminary assessment of the consistency of combining ability under environmental variability. However, the extent to which these specific lines contribute differently to hybrid performance under tropical environments remains underexplored.

This study therefore aimed to (i) evaluate the combining ability of 20 Indonesian sweet corn inbred lines and four testers across contrasting wet and dry seasons, and (ii) apply GGE biplot analysis to identify superior parents and hybrids. By integrating line × tester analysis with GGE biplot visualization, this research provides novel insights into tropical sweet corn breeding, filling a critical knowledge gap in environments characterized by seasonal variability.

## Materials & Methods

### Plant material and experimental design

This study utilized 20 inbred sweet corn lines (L) and four testers (T), resulting in 80 cross combinations following a line × tester mating scheme (Table 1). All parental lines were developed at the Plant Breeding Laboratory, Faculty of Agriculture, Universitas Padjadjaran, and were previously characterized for high yield potential and resistance to downy mildew. Several lines had also been evaluated under intercropping with chili pepper (Ruswandi et al., 2016) and were reported to exhibit stable adaptability using AMMI analysis (Ruswandi et al., 2020). The materials showed broad phenotypic variability for yield-related traits, making them suitable candidates for hybrid development. The four testers used in this study were elite sweet corn inbred lines, not commercial hybrids, developed within the Universitas Padjadjaran breeding program. These testers were selected based on their contrasting agronomic performance and use as standard male parents in previous combining ability evaluations.

**Table 1:** List of 20 inbred lines and four testers of sweet corn (*Zea mays saccharata* L.) used in the line × tester mating design. Each line was derived from single crosses, selfed, and selected for uniformity, while testers were elite commercial hybrids.

**No.**	**Code**	***Line*** **s**
1	L1	Line derive from single cross SR 4 and SRBonz
2	L2	Line derive from single cross SR 4 and SRTaln
3	L3	Line derive from single cross SR 17 and SRJamb
4	L4	Line derive from single cross MSR 17.6.7 and SRBonz
5	L5	Line derive from single cross MSR 17.6.7 and SRLatz
6	L6	Line derive from single cross MSR 17.6.7 and SRTaln
7	L7	Line derive from single cross MSR 25.5.1 and SR 17
8	L8	Line derive from triple cross MSR 17.2.3 and SR 17 and SR Bonz
9	L9	Line derive from triple cross MSR 17.2.3 and SR 17 and SR Latz
10	L10	Line derive from triple cross MSR 17.2.3 and SR 17 and Taln
11	L11	Line derive from triple cross MSR 17.6.7 and SR 4 and M6SR 17.2.3
12	L12	Line derive from triple cross MSR 17.6.7 and SR 4 and M6SR 17.6.7
13	L13	Line derive from triple cross MSR 25.5.1 and SR 17 and SR 4
14	L14	Line derive from SRSB
15	L15	Line derive from SRBonz
16	L16	Line derive from SRJamb
17	L17	SRTaln
18	L18	SRSB
19	L19	SRTHI-2
20	L20	SRTHI-3
21	T1	SRBonz
22	T2	SRJamb
23	T3	SRTaln
24	T4	SRSB

### Hybridization procedure

Hybrid seed production was conducted under field conditions at the Universitas Padjadjaran experimental station prior to yield evaluation. All parental inbred lines and testers were grown in isolated blocks to prevent pollen contamination. Controlled crosses were performed following standard hand-pollination procedures in maize. Female parents (lines) were detasseled before anthesis to avoid self-pollination, while tassels of male parents (testers) were bagged prior to pollen shedding. At silking, ears of the female plants were covered with paper bags, and pollen collected from the designated tester was manually applied to the exposed silks. Pollinated ears were rebagged immediately and labeled according to the specific line × tester combination. Mature ears were harvested at physiological maturity, dried, shelled, and stored under controlled conditions until use in field evaluation trials.

### Field experimental and data collection

The experiments were carried out in Garut Regency, West Java Province (1,346 m above sea level; see Tables 2 and 3 for site conditions). Trials were conducted in two contrasting growing seasons: April–July 2023 (dry season) and December 2023–March 2024 (wet season). A randomized complete block design (RCBD) with three replications was used. Each plot consisted of a single row of 5 m length, with a planting distance of 75  × 25 cm (approximately 26–28 plants per plot). Grain yield per hectare was extrapolated from plot fresh ear weight using the conversion factor based on plant population density (53,333 plants ha^−^^1^).

Ear traits, including ear diameter, ear length, kernel row number, and ear weight, were measured using standard post-harvest procedures. Ear diameter was recorded at the midsection of the cob using a digital caliper, while ear length was measured without husk. Plant biomass included the fresh weight of the whole plant (stem + leaves + ears). Sweetness (brix) was measured using a handheld refractometer after grinding the kernels into homogenized extract.

### Data analysis

Line × Tester ANOVA was performed using AGD-R software (version 5.1), following the method recommended by Singh & Chaudary (1979). GGE biplots were generated using GEA-R (version 4.1), following the singular value partitioning method recommended by Yan & Kang (2002). The model was expressed as Yan & Kang (2002):

\begin{eqnarray*}{Y}_{ij}-{\beta }_{j}={\lambda }_{1}{\xi }_{i1}{\eta }_{j1}+{\lambda }_{2}{\xi }_{i2}{\eta }_{j2}+{}_{ij} \end{eqnarray*}

where, Y_*ij*_: genotype value of the cross between lines ith dan tester jth, *β*_*j*_: the average value of crosses involving tester jth, *λ*_1_ dan *λ*_2_= the single value for PC1 and PC2, *ξ*_*i*1_*η*_*j*1_: the eigenvector for PC1 associated with lines ith, *ξ*_*i*2_*η*_*j*2_: the eigenvector for PC2 associated with lines jth, *ɛ*_*ij*_: the effect of experimental error on the traits ith and traits jth.

The GGE biplot was used in three forms:

1. Mean *vs.* Stability—to identify stable high-performing parents.
2. Discriminativeness *vs.* Representativeness—to evaluate tester efficiency.
3. Which-Won-Where/What—to detect superior hybrid combinations.

These graphical interpretations allowed simultaneous assessment of GCA, SCA, and genotype × environment interactions, facilitating the identification of promising parental lines and hybrids.

### Data availability

The complete raw dataset of all measured traits across two contrasting seasons has been deposited as a Supplemental File (‘Raw Data—Sweet Corn Hybrids.xlsx’) in accordance with PeerJ’s open data policy. The dataset includes agronomic and quality traits for 80 hybrid combinations evaluated in Garut, Indonesia, across wet and dry seasons.

**Table 2:** Monthly rainfall during the two cropping seasons in Garut Regency, Indonesia. Season 1 was conducted from April to July 2023, and Season 2 from December 2023 to March 2024. Data were obtained from the Garut Regency Agriculture Office.

**Cropping season**	**Location**	**Rainfall (mm)**				
Season 1	Cikajang Village, Cikajang District, Garut Regency, Coordinate (7,3569°S, Longitude 107.8069°E)	April	May	June	July	Average
		373.5	176.5	233.5	332.5	279
Season 2		December	January	February	March	Average
		397.5	295	323	230.5	311.5

**Table 3:** Soil physicochemical properties at the experimental site in Garut Regency, Indonesia. Parameters were analyzed at the Soil Chemistry and Plant Nutrition Laboratory, Universitas Padjadjaran. Criteria classification follows Indonesian soil fertility standards.

**No**	**Parameter**	**Result**	**Criteria**
1	pH	5,47	Strongly Acid
2	Organic –C	6, 70%	Very High
4	Total –N	0, 74%	High
5	P_2_O_5_	75,48 ppm P	Poor
6	K_2_O	19,71 mg/100g	Low
7	**Texture**		
	Sand	48%	*Loam*
	Silt	40%	
	Clay	12%	

## Results

### Genotype × Environment Interaction

Analysis of variance of the line × tester crosses across two contrasting seasons is presented in Table 4. The analysis of variance (ANOVA) revealed highly significant effects (*p* < 0.01) for genotypes, lines, testers, and their interactions across all traits, including ear diameter, ear length, kernel rows per ear, ear weight, plant biomass, brix, and yield. Seasonal effects were also significant for most traits, confirming strong environmental influence on sweet corn performance. In addition, significant season × genotype, season × line, and season × line × tester interactions indicated the presence of genotype × environment interaction (GEI).

**Table 4:** Mean squares from analysis of variance (ANOVA) for yield and yield-related traits in sweet corn hybrids evaluated across two contrasting seasons. Genotypes were partitioned into lines, testers, and line × tester interactions.

Source of variation	Df	ED	EL	NR	EW	PB	Brix	Yield
Season	1	6.52^*^	2.50	1.00	7.33^**^	8,582.37^**^	8.71^**^	13.08^**^
Rep(Season)	4	1.52	4.94^**^	3.10^*^	3.82^**^	6.58^**^	2.82^*^	3.08^*^
Genotypes	79	2.92^**^	1.65^**^	2.13^**^	2.47^**^	2.67^**^	2.18^**^	3.41^**^
Line	19	4.12^**^	1.48	2.05^**^	2.05^**^	2.29^**^	1.87^*^	3.09^**^
Tester	3	5.08^**^	3.27^*^	9.59^**^	5.44^**^	6.27^**^	2.67^*^	16.17^**^
Line:Tester	57	2.41^**^	1.62^**^	1.77^**^	2.45^**^	2.61^**^	2.25^**^	2.84^**^
Season:Genotypes	79	1.91^**^	1.61^**^	1.50^**^	1.81^**^	2.18^**^	2.20^**^	2.18^**^
Season:Line	19	2.28^**^	1.55	0.96	2.17^**^	2.42^**^	1.21	1.94^*^
Season:Tester	3	0.20	0.61	1.01	1.57	1.81	2.56	3.57^*^
Season:Line:Tester	57	1.88^**^	1.68^**^	1.71^**^	1.71^**^	2.12^**^	2.51^**^	2.18^**^

**Notes.**

ED ear diameter EL ear length NR number of rows per ear EW ear weight PB plant biomass

* Significance at *p* < 0.05.

** Significance at *p* < 0.01.

### Genetic variance and heritability

Component analysis showed that dominance variance exceeded additive variance across all measured traits, suggesting that hybrid performance was strongly influenced by non-additive genetic effects (Table 5). Broad-sense heritability estimates were moderate to high (0.47–0.66), whereas narrow-sense heritability was very low (0.01–0.13), pointing to a greater role of dominance and epistatic interactions in shaping hybrid outcomes.

**Table 5:** Estimates of variance components, genetic parameters, and heritability for yield and yield-related traits in sweet corn across seasons. Values represent additive variance, dominance variance, degree of dominance, and broad-sense heritability (H^2^).

Variance estimate	ED	EL	NR	EW	PB	Brix	Yield
Genotype variance	265.45	53.36	31.20	104,956.50	159,758.70	39.70	300.41
Line variance	0.49	0.00	0.01	0.01	0.01	0.01	0.07
Tester variance	0.15	0.03	0.07	80.50	138.38	0.01	0.74
LinexTester variance	1.62	0.25	0.14	780.91	1,218.36	0.29	2.06
GCA variance	0.34	0.01	0.04	5.53	27.36	0.01	0.37
Additive variance	1.37	0.03	0.16	22.13	109.43	0.01	1.47
Dominance variance	6.48	1.02	0.57	3,123.63	4,873.45	1.16	8.22
Environmental variance	4.30	1.16	0.46	1,851.03	3,438.13	1.06	5.05
Broad heritability	0.65	0.47	0.61	0.63	0.59	0.52	0.66
Narrow heritability	0.11	0.01	0.13	0.01	0.01	0.01	0.10

**Notes.**

ED ear diameter EL ear length NR number of rows per ear EW ear weight PB plant biomass

### Combining ability and hybrid superiority

**Figure 1:**
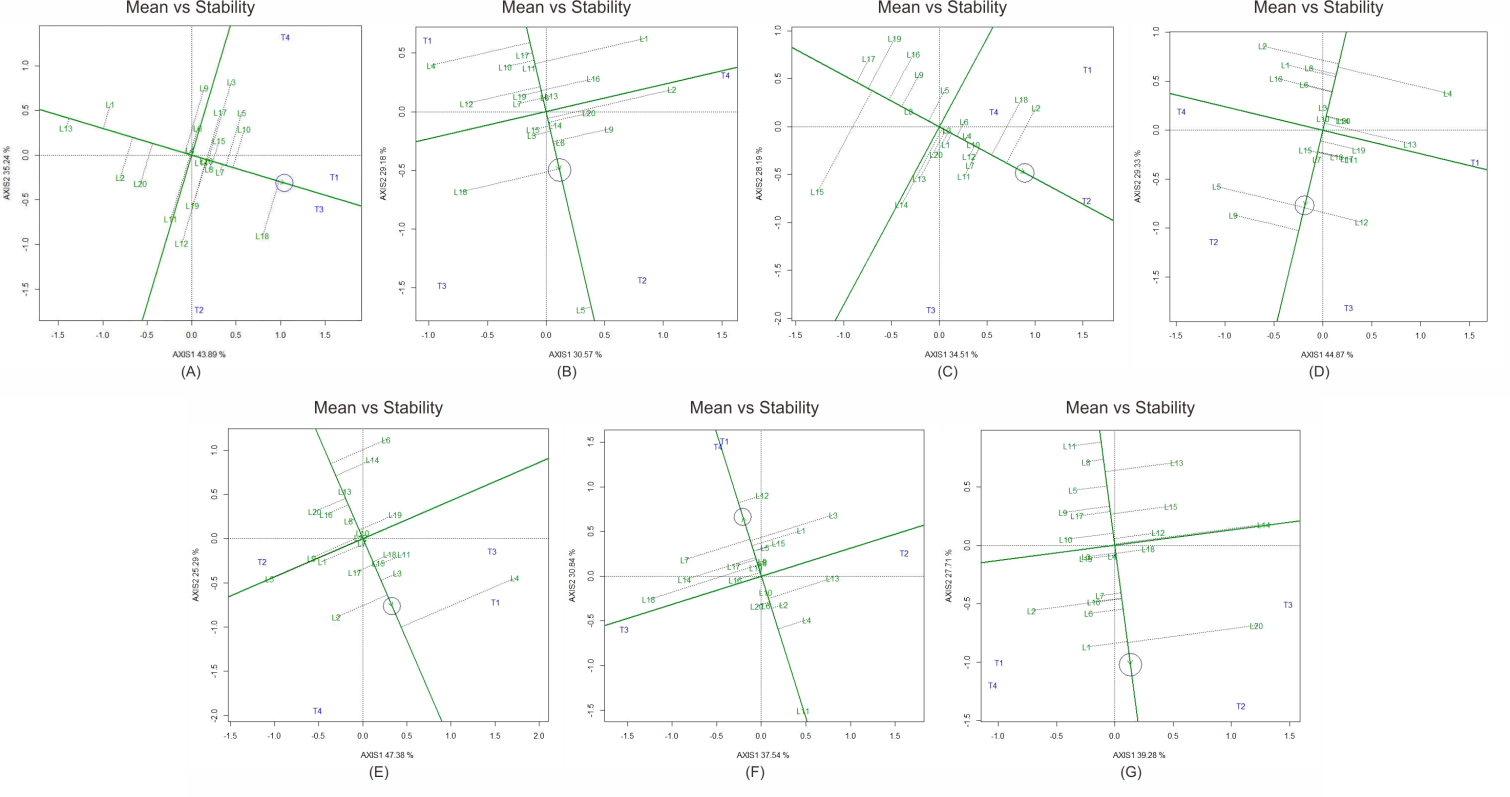
GGE biplot of general combining ability (GCA) effects of 20 sweet corn lines and 4 testers for yield and yield-related traits across two contrasting seasons. The biplot identifies superior parental lines for specific traits. (A) Ear diameter, (B) ear length, (C) kernel rows per ear, (D) ear weight, (E) plant biomass, (F) brix, and (G) yield. Genotypes located closer to the ideal point demonstrate both high mean performance and stability across environments.

The GGE biplot “mean *vs.* stability” analysis (Fig. 1) identified several inbred lines with consistent combining ability across environments. Line L18 emerged as the best general combiner for ear diameter and ear length, L2 excelled in kernel row number, L9 in ear weight, L4 in biomass, L12 in brix, and L20 in yield. Their proximity to the ideal genotype position in the biplot confirmed strong and stable GCA effects.

**Figure 2:**
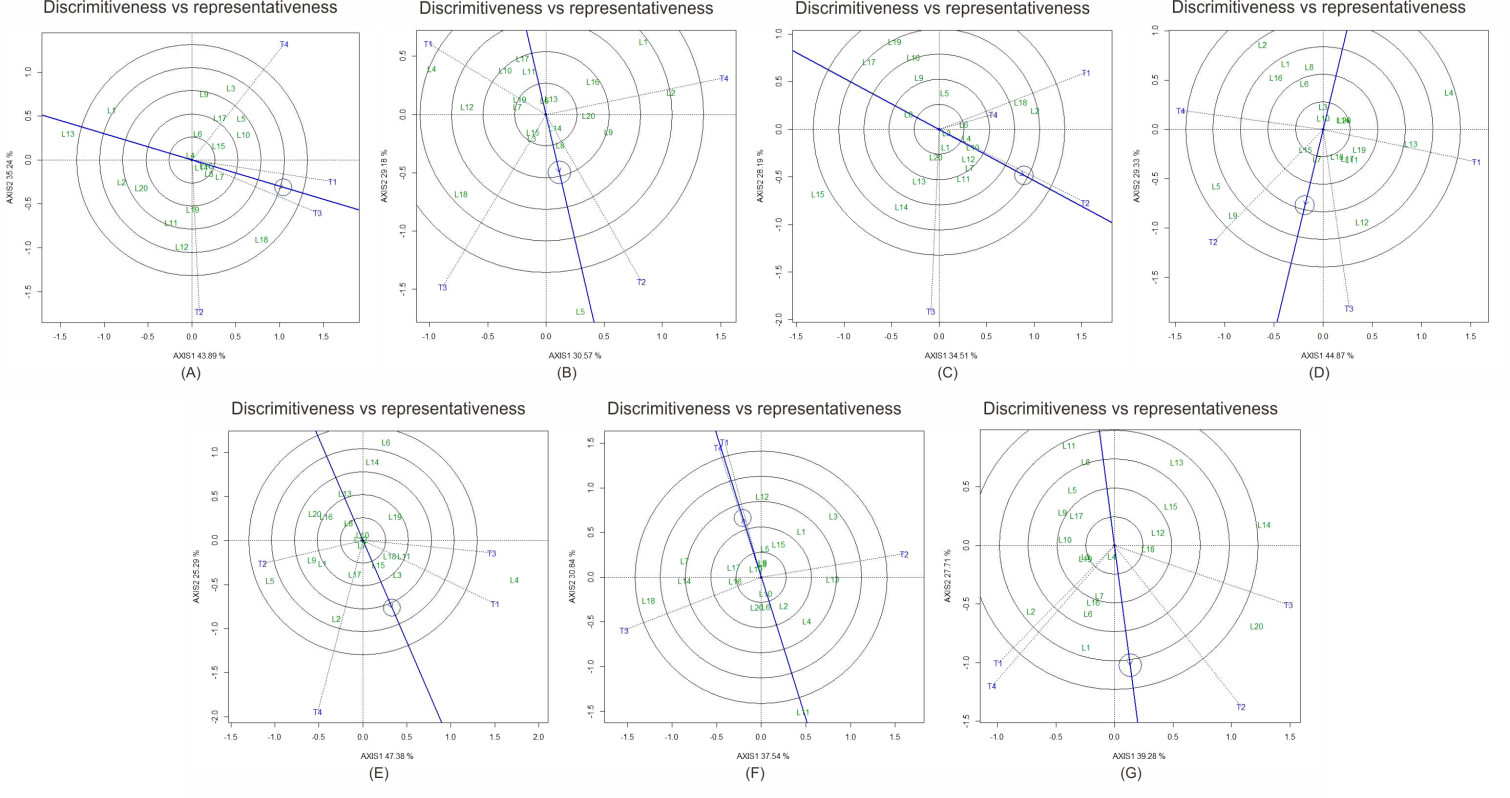
GGE biplot “Discriminativeness *vs.* Representativeness” showing tester efficiency in evaluating sweet corn lines for seven traits. (A) Ear diameter, (B) ear length, (C) kernel rows per ear, (D) ear weight, (E) plant biomass, (F) brix, and (G) yield. Testers with long vectors and proximity to the average tester coordinate (ATC) axis are considered ideal.

Tester performance was also discriminated effectively. T3 served as the best tester for ear diameter and ear weight, T2 for ear length and kernel rows per ear, and T1 for brix (Fig. 2). Testers with long vectors located near the average coordinate were both discriminative and representative, making them suitable for evaluating new parental lines.

**Figure 3:**
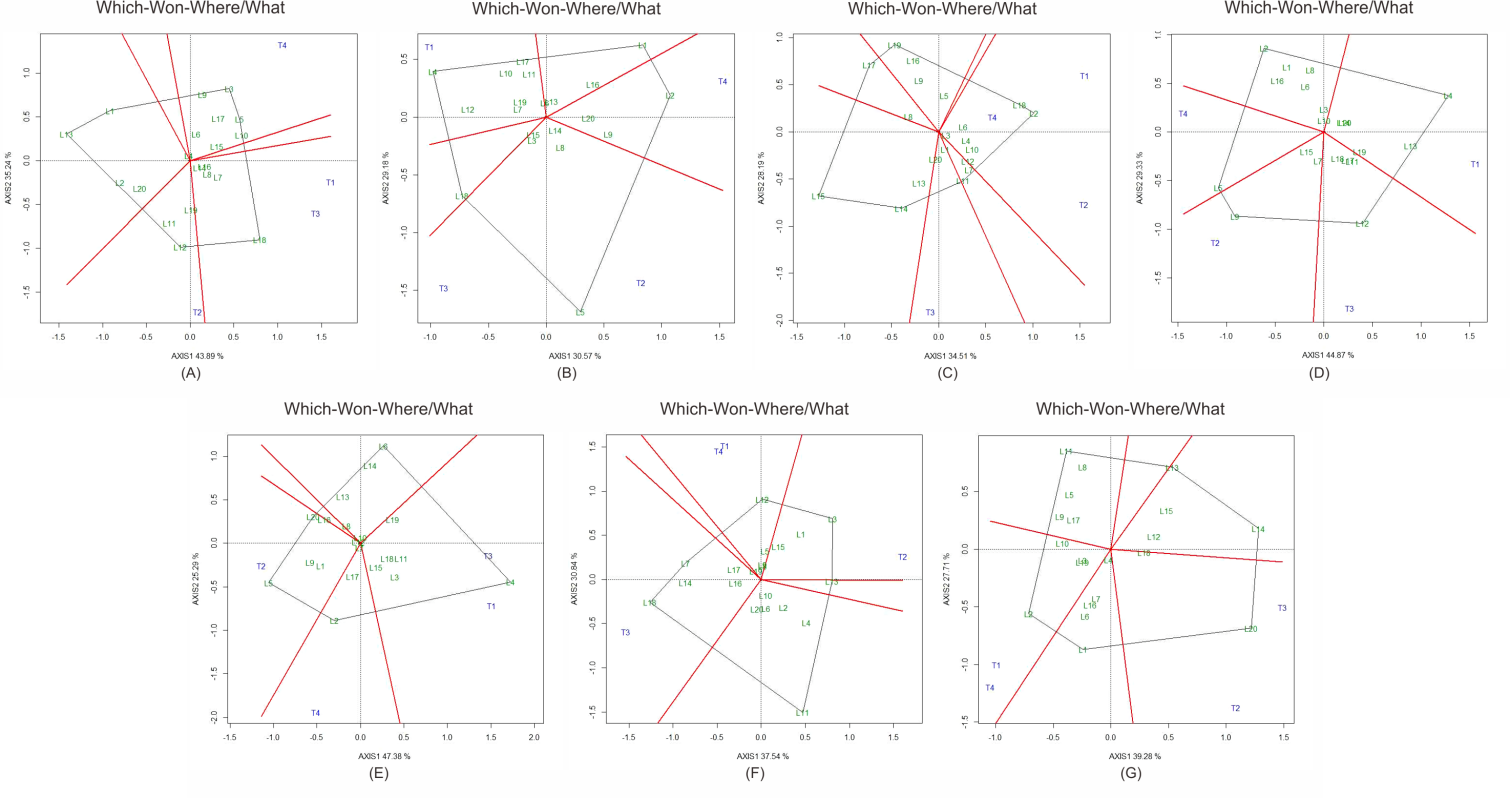
“Which-won-where” GGE biplot showing the partitioning of sweet corn hybrids into sectors across contrasting environments. Each sector identifies hybrids with the best performance in specific trait–environment combinations. (A) Ear diameter, (B) ear length, (C) kernel rows per ear, (D) ear weight, (E) plant biomass, (F) brix, and (G) yield. Hybrids located at polygon vertices represent the best-performing cross combinations in each sector.

The “which-won-where/what” biplot (Fig. 3) further highlighted hybrids with strong SCA effects. L2  × T4 consistently outperformed for ear length, kernel row number, biomass, and yield, while L5  × T4 was superior in ear diameter and weight. L12  × T1 showed the highest brix content. For yield, L20  × T3 and L2  × T4 repeatedly occupied the winning sectors across seasons, validating their potential as stable, high-yielding hybrids.

Collectively, these results indicate that both GCA and SCA play crucial roles in sweet corn improvement, with several parental lines (L18, L2, L9, L4, L12, and L20) and testers (T1, T2, T3) emerging as reliable combiners, while hybrids such as L2  × T4, L5  × T4, and L12  × T1 stood out as promising candidates for further evaluation.

Because only two seasonal environments were evaluated at a single location, the stability patterns observed in the GGE biplots should be interpreted as consistency across contrasting seasonal conditions rather than definitive multi-environment stability.

## Discussion

### Genotype × Environment interaction

Yield stability in sweet corn was substantially shaped by genotype × environment interactions under contrasting seasonal conditions. Fluctuating rainfall, variable soil fertility, and temperature shifts typical of tropical systems strongly affected yield outcomes. Similar results have been reported in maize and other cereals, where G × E was a major contributor to yield inconsistency across seasons and sites (Crossa, 1990; Fan et al., 2007; Wicaksana et al., 2022).

### Genetic variance and heritability

Variance component analysis revealed that dominance effects outweighed additive ones, confirming the central role of non-additive genetic mechanisms in determining productivity. This aligns with classical theory suggesting that heterosis in cross-pollinated crops like maize is largely driven by dominance and epistasis (Barh et al., 2015; Angui et al., 2020).

The extremely low narrow-sense heritability estimates (*h*^2^ ≈ 0.01–0.13) observed in this study indicate that additive genetic variance contributed minimally to the expression of key agronomic traits. Several biological and methodological factors may explain this pattern. First, the inbred lines used in this study originated from the same long-term breeding program, which may have resulted in partial relatedness and reduced additive variance. Second, dominance and epistatic interactions may have masked additive gene effects, especially under contrasting seasonal conditions where stress responses can amplify non-additive genetic expression. Third, using a limited number of environments (two seasons) restricts the precision of variance component estimation, potentially inflating dominance estimates relative to additive variance. Similar observations have been reported in tropical maize populations where heterosis is pronounced and additive effects are less detectable (Bänziger & Cooper, 2001; Labroo, Studer & Rutkoski, 2021). Therefore, the results align with expectations for cross-pollinated crops but should be interpreted with caution.

### Combining ability and hybrid superiority

General combining ability (GCA) estimates identified several lines with consistent additive contributions. Lines such as L18, L2, L9, L4, L12, and L20 performed reliably across traits and seasons, supporting their role as strong parents for hybrid breeding. Positive GCA values have been consistently associated with favorable additive effects in maize and related crops (Badu-Apraku et al., 2021; Awad-allah et al., 2022). The GGE biplot “mean *vs.* stability” analysis further confirmed these lines as both high-performing and stable, emphasizing their suitability as elite parents for tropical environments.

The use of the GGE biplot “mean *vs.* stability” analysis effectively distinguished superior general combiners, not only based on mean performance but also on stability across environments (Yan & Tinker, 2006; Momeni et al., 2020). Such findings highlight the importance of carefully selecting parental lines with strong GCA effects, as these lines form the genetic foundation for developing high-yielding and widely adapted sweet corn hybrids in tropical environments.

Specific combining ability (SCA) highlighted crosses that benefited from non-additive interactions. Hybrids such as L2  × T4 (ear length, kernel rows, biomass, yield), L5  × T4 (ear diameter, weight), and L12  × T1 (brix) demonstrated strong and consistent SCA effects across seasons. Reports in maize and sorghum similarly show that hybrids with high SCA often surpass their parental means due to favorable dominance and epistatic interactions (Ruswandi et al., 2015a; Ruswandi et al., 2015b; Sunny et al., 2022). Interestingly, favorable SCA was sometimes produced by crosses between parents with contrasting GCA values, reflecting the complex genetic architecture of heterosis (Shumbulo, Nigussie & Alamerew, 2018; Lu et al., 2020).

The effectiveness of the “which-won-where/what” biplot in distinguishing superior hybrid combinations reinforces its value as a practical breeding tool. Comparable applications in sunflower, sorghum, and maize confirm its reliability for hybrid identification (Ahmed et al., 2019; Kannababu et al., 2017; Shojaei et al., 2022). In this study, hybrids such as L2  × T4 and L20  × T3 emerged as strong candidates for multilocation trials and potential release.

By integrating line × tester analysis with GGE biplot visualization, this research offered a comprehensive framework for assessing both additive and non-additive genetic contributions to sweet corn performance. The identification of elite parents and high-performing hybrids provides a practical roadmap for breeding programs in Indonesia, where seasonal variability and environmental heterogeneity pose major challenges.

### Limitations of stability assessment

Although GGE biplot analysis allows visualization of genotype performance across environments, the use of only two seasonal datasets limits the robustness of stability conclusions. The patterns observed here should therefore be regarded as preliminary indicators of consistency across contrasting seasonal conditions rather than comprehensive stability across multiple agroecological zones. Future multi-location, multi-year trials are necessary to confirm the stability of the identified hybrids.

The predominance of non-additive effects indicates that hybrid breeding should be prioritized over pure-line selection to fully exploit heterosis. Furthermore, the identification of discriminative and representative testers (T1, T2, and T3) provides practical guidance for future breeding programs to efficiently evaluate new inbred lines (Hallauer & Miranda, 2010; Maazou et al., 2022). Collectively, these findings contribute to establishing a sustainable breeding pipeline for high-yielding and stable sweet corn hybrids in tropical environments.

From a practical standpoint, the development of the identified superior hybrids holds promise not only for boosting farmer incomes but also for strengthening sweet corn’s role as a high-value crop in both domestic and export markets (Swapna, Jadesha & Mahadevu, 2020; Baveja et al., 2022).

## Conclusions

This study demonstrated that non-additive genetic effects were predominant in determining hybrid performance, emphasizing the importance of heterosis-based breeding in tropical sweet corn. Several parental lines (L18, L2, L9, L4, L12, and L20) and hybrids (particularly L2  × T4 and L20  × T3) showed consistent performance across contrasting seasonal conditions. While these findings provide valuable direction for hybrid development, the observed performance consistency across seasons should be regarded as preliminary and requires validation through broader multi-location testing. The combined use of Line × Tester analysis and GGE biplot proved effective for identifying promising parents and hybrids and can support future breeding strategies under tropical environments.

##  Supplemental Information

10.7717/peerj.20966/supp-1 Supplemental Information 1 Raw data of sweet corn line × tester hybrids evaluated across two seasons in Garut, Indonesia ED Ear Diameter; EL Ear Length; NoR Number of Row ; EW Ear Weight; BIO Biomass; BRIX Brix ; Yield Yield

## Additional Information and Declarations

### Data Availability

The following information was supplied regarding data availability: The data is available at Mendeley: Syihab, Fakhri Nasharul; Maulana, Haris; Ismail, Ade; Maxiselly, Yudithia; Fauzan, Mochamad Rafi; Abdullah, Moh. Ali; Karuniawan, Agung ; Yuwariah, Yuyun; Ruswandi, Dedi (2024), “Dataset For Combining Ability of Earliness and Yield in Sweet Corn Using GGE Biplot”, Mendeley Data, V2, doi: 10.17632/5dkk9gjsth.2. The raw measurements are available in the Supplemental File.
